# Acute Kidney Injury and High-Sensitivity Cardiac Troponin T Levels in the Emergency Department

**DOI:** 10.1001/jamanetworkopen.2024.19602

**Published:** 2024-08-30

**Authors:** Love Cyon, Erik Kadesjö, Gustaf Edgren, Andreas Roos

**Affiliations:** 1 Department of Medicine, Clinical Epidemiology Division, Karolinska Institutet, Solna, Stockholm, Sweden; 2 Department of Emergency and Reparative Medicine, Karolinska University Hospital, Huddinge, Stockholm, Sweden; 3 Department of Cardiology, Södersjukhuset, Stockholm, Sweden

## Abstract

**Importance:**

The clinical implications of high-sensitivity cardiac troponin T (hs-cTnT) measurements in patients with acute kidney injury (AKI) in the emergency department (ED) are largely unknown.

**Objectives:**

To investigate associations between serum creatinine (SCr) concentrations and hs-cTnT kinetics, as well as the clinical accuracy of hs-cTnT for myocardial infarction (MI) in patients with AKI.

**Design, Setting, and Participants:**

This retrospective cohort study included 15 111 patient visits to 7 EDs in Sweden from December 9, 2010, to August 31, 2017, by patients 18 years or older fulfilling AKI criteria with 2 or more SCr measurements and 1 or more hs-cTnT measurement. Statistical analysis was performed from October 2, 2022, to September 28, 2023.

**Exposure:**

Dynamic change in SCr during the ED visits.

**Main Outcomes and Measures:**

Linear mixed-effects models were used to estimate the log-linear regression of kinetic change in hs-cTnT. Logistic regression models were applied to calculate odds ratios (ORs) for change in hs-cTnT indicating acute myocardial injury (Δhs-cTnT >20% and elevated hs-cTnT >14 ng/L) in association with change in SCr, and to assess the diagnostic performance of hs-cTnT for MI in patients with chest pain.

**Results:**

There was a total of 15 211 visits by 13 638 patients (median age, 74 years [IQR, 64-83 years]; 8709 men [57%]), of whom 1174 (8%) had an MI. Overall, 11 353 of patients at 14 037 visits without an MI diagnosis (81%) had myocardial injury, and 4396 patients at 14 037 visits (31%) had acute myocardial injury. The change in hs-cTnT among patients without MI was 1.8-fold higher in the highest vs the lowest change in SCr quartile (64.7% [95% CI, 58.4%-71.5%] vs 36.3% [95% CI, 32.4%-40.7%]; exponentiated β coefficient, 1.78 [95% CI, 1.62-1.96]). Patients in the former group were twice as likely to have acute myocardial injury (39% [1378 of 3516 visits] vs 23% [817 of 3507 visits]; adjusted OR, 2.32 [95% CI, 2.08-2.59]). Using a 0 hours hs-cTnT cutoff value of 52 ng/L or higher assigned 627 of 2388 patients (26%) with chest pain to a high-risk group in whom the specificity and positive predictive value for MI was low (78.5% [95% CI, 76.7%-80.2&] and 27.6% [95% CI, 24.1%-31.3%], respectively).

**Conclusions and Relevance:**

This cohort study of patients in the ED suggests that dynamic change in SCr among patients with AKI was associated with hs-cTnT concentrations indicative of acute myocardial injury. These observations were accompanied by poor performance of recommended hs-cTnT–based algorithms for MI risk stratification.

## Key Points

**Question:**

What are the clinical implications of high-sensitivity cardiac troponin T (hs-cTnT) measurements in patients with acute kidney injury (AKI)?

**Findings:**

In this cohort study of 15 111 emergency department visits by 13 638 patients with AKI, hs-cTnT concentrations indicative of acute myocardial injury were evident in almost 1 of 3 visits without myocardial infarction (MI). In addition, hs-cTnT–based algorithms for identifying patients at high risk for MI .showed poor performance.

**Meaning:**

Findings of this study suggest that dynamic hs-cTnT concentrations are commonly observed in patients with AKI, limiting the clinical accuracy for MI, and highlights the need for novel hs-cTn–based approaches to increase specificity for MI among this patient population.

## Teaser

This cohort study investigates myocardial injury and the association between serum creatinine concentrations and high-sensitivity cardiac troponin T kinetics, as well as the clinical accuracy of high-sensitivity cardiac troponin T for myocardial infarction in patients with acute kidney injury.

## Introduction

High-sensitivity cardiac troponin (hs-cTn) assays have been used for the diagnosis of myocardial infarction (MI) in emergency departments (EDs) in Europe for more than 10 years and have improved early and low-end MI diagnostics.^1,2,3^ An MI diagnosis relies on the presence of acute myocardial injury, which is defined as a typical increase and/or decrease of elevated hs-cTn concentrations.^4^

Acute kidney injury (AKI) occurs in many different clinical settings and affects approximately 20% of hospitalized patients.^5,6,7^ Several studies have investigated the association between serum creatinine (SCr) and hs-cTnT in patients with chronic kidney disease (CKD),^8,9,10,11,12^ but knowledge about the association between variation of kidney function—as ascertained using serial SCr measurements and dynamic hs-cTn changes consistent with acute myocardial injury—in AKI is limited.^13,14,15,16,17^ Evidence about AKI and the diagnostic performance of hs-cTn–based MI risk stratification algorithm criteria in the ED is also sparse.^3^ Therefore, using a large observational cohort study, we investigated the prevalence of myocardial injury and the association between SCr and hs-cTnT kinetics among patients in the ED with AKI, as well as the clinical accuracy of hs-cTnT measurements for MI in patients presenting with AKI and chest pain.

## Methods

### Data Sources

Data collection for the study occurred at 7 EDs in Sweden from December 9, 2010, to August 31, 2017. Data were collected from each hospital’s medical record system to identify all ED visits among patients 18 years of age or older. Laboratory data were obtained from the local clinical chemistry databases. The Elecsys 2010 system (Roche Diagnostics) was used for hs-cTnT analyses at all hospitals.^18^ Data were thereafter sent to the Swedish National Board of Health and Welfare, for linkage with information on comorbidities and discharge diagnoses from the National Patient Register, information on medications from the Prescribed Drug Register, and information on cause-specific mortality from the Cause of Death Register.^19,20,21^ Further data on MI diagnoses and coronary interventions were retrieved from the Swedish Web System for Enhancement and Development of Evidence-Based Care in Heart Disease Evaluated According to Recommended Therapies (SWEDEHEART) register.^22^ The Swedish Ethical Review Authority approved this study, which was conducted using deidentified data. The approval did not require patient consent for study inclusion, as Sweden allows for research to take place with waived consent in limited circumstances. The study adhered to the principles of the Declaration of Helsinki^23^ and the reporting of this observational cohort study followed the Strengthening the Reporting of Observational Studies in Epidemiology (STROBE) reporting guideline.^24^

### Study Population and Definition of AKI

All visits by patients in the study base with 2 or more SCr measurements during the visit were eligible (eFigure 1 in Supplement 1). Acute kidney injury was defined according to the Kidney Disease Improving Global Outcomes (KDIGO) criteria^6^ as any of the following: (1) an increase or decrease in SCr by 0.3 mg/dL or more (to convert to micromoles per liter, multiply by 88.4) within 48 hours during the visit, or (2) an increase in SCr of 1.5 or more times the most recently registered value within 7 days or a corresponding relative increase between any SCr measured during the visit, or (3) a decrease in SCr with the highest SCr being 1.5 or more times higher than the lowest SCr measured during the visit, or (4) a diagnosis of AKI according to the *International Statistical Classification of Diseases and Related Health Problems, Tenth Revision* (*ICD-10*) codes registered at the hospital visit (*ICD-10* code N17) (eTable 1 in Supplement 1). All visits by patients fulfilling criteria for AKI and who had 1 or more hs-cTnT measurement were subsequently included. Visits by patients with ongoing dialysis treatment were excluded. Analyses on the diagnostic performance of hs-cTnT measurements for MI were conducted for all visits by patients presenting with chest pain to the ED after excluding patients with ST-segment elevation MI (STEMI) and those who fulfilled AKI criteria only after coronary angiography was conducted (eFigure 2 in Supplement 1).

### Definition of Biochemical Measurements and Comorbidities

Categorization of AKI was defined according to the KDIGO criteria (eTable 1 in Supplement 1).^6^ The relative change in SCr levels was defined as the relative change between the highest and lowest SCr level during the visit. The change in SCr velocity was defined as the change in SCr per hour, using all recorded SCr measurements during the visit. Similar measurements were computed for hs-cTnT. For velocity computations, only sample pairs measured 3 hours or less from each other were used. Myocardial injury was defined as any hs-cTnT level greater than 14 ng/L (to convert to micrograms per liter, multiply by 0.001) and acute myocardial injury as any hs-cTnT level greater than 14 ng/L with a relative change in hs-cTnT level of greater than 20%.^4^ An acute MI at the visit was defined according to primary discharge diagnoses (*ICD-10* codes I21 or I22), and/or a concurrent MI diagnosis registered in SWEDEHEART. STEMI diagnoses were ascertained by registrations in SWEDEHEART. Ongoing medication was defined as 2 or more dispensed medications in the year preceding the visit. Estimated glomerular filtration rate (eGFR) at presentation was calculated using the Chronic Kidney Disease Epidemiology Collaboration equation.^25^

### Statistical Analysis

Statistical analysis was performed from October 2, 2022, to September 28, 2023. Logistic regression models were used to estimate odds ratios (ORs) with robust SEs for the occurrence of acute myocardial injury in patients without MI in association with quartiles of change in SCr during the visit. Models were adjusted for the following covariates: eGFR at presentation, patient age, patient sex, and for a previous diagnosis of MI, heart failure, atrial fibrillation, stroke, diabetes, or chronic obstructive pulmonary disease. Correlation between eGFR at presentation and first hs-cTnT in patients without MI was evaluated with the use of Pearson rank correlation.

A linear mixed-effects model with random intercept was used to estimate the association between quartiles of change in SCr and adjusted differences in the geometric mean change in hs-cTnT in patients without MI. The hs-cTnT concentrations were log-transformed to account for nonlinear distribution. The models were adjusted for all variables used previously. The exponentiated β coefficient (exp[β]) could be interpreted as the multiplicative difference in the geometric mean of the estimated change in hs-cTnT, comparing the different quartiles of change in SCr. A linear mixed-effects model was also fitted to assess the change in hs-cTnT velocity as a function of the change in SCr velocity, with both measures being log-transformed before modeling.

The diagnostic performance of hs-cTnT for an MI diagnosis with the following measures were evaluated: (1) absolute 0 hours hs-cTnT (ie, first hs-cTnT), (2) absolute change in hs-cTnT, and (3) relative change in hs-cTnT between the first and second hs-cTnT concentrations, of which the 2 latter analyses were restricted to visits with a second hs-cTnT recorded within 6 hours from the first hs-cTnT. Cutoff values for the MI diagnosis were assessed according to the following metrics of the receiver operating characteristic curve: the Youden J-index, a sensitivity of 90% or more, and a specificity of 90% or more. The clinical accuracy was further calculated for a cutoff at 15 ng/L (the 99th percentile upper reference limit), at 52 ng/L (the European Society of Cardiology [ESC] rule-in criterion^3^), and at 20% relative change in hs-cTnT. The performance of the hs-cTnT–based 0-/1-hour MI risk stratification algorithm criteria recommended by the current ESC guidelines were also evaluated.^3^ Statistical analyses were conducted using SAS Statistical Analysis Software 11, version 9.4 (SAS Institute Inc).

## Results

### Study Population

Of a total of 3 383 209 visits within the cohort, the study population included 15 211 visits by 13 638 patients (median age, 74 years [IQR, 64-83 years]; 8709 men [57%] and 6502 women [43%]) with AKI and 1 or more hs-cTnT measurement (Table 1). Altogether, 1174 visits (8%) had a diagnosis of MI associated with the visit. Patients with MI were less likely to be female and to have a previous diagnosis of heart failure and atrial fibrillation, but more likely to have had a prior MI.

**Table 1.** Patient Characteristics

Characteristic	Visits without MI diagnosis, No. (%)	Visits with MI diagnosis, No. (%)
No. of visits (row %)	14 037 (92)	1174 (8)
No. of unique patients	12 551 (89)	1087 (93)
Age, median (IQR), y	74 (64-83)	76 (66-85)
Sex		
Female	6072 (43)	430 (37)
Male	7965 (57)	744 (63)
Comorbidities		
Prior MI	2375 (17)	439 (28)
Prior revascularization	2069 (15)	268 (23)
Prior stroke	2080 (15)	152 (13)
Heart failure	4477 (32)	294 (25)
Diabetes	3632 (26)	370 (32)
Hypertension	8063 (57)	729 (62)
Atrial fibrillation	4202 (30)	238 (20)
COPD	2390 (17)	129 (11)
Laboratory data		
First hs-cTnT concentration, median (IQR), ng/L	33 (16-66)	100 (39-7810)
No. of hs-cTnT measurements, median (IQR)	2 (1-3)	3 (3-5)
No. of visits with ≥2 hs-cTnT measured	8937 (64)	1126 (96)
Peak hs-cTnT concentration, median (IQR), ng/L	40 (19-86)	846 (221-2717)
Presence of myocardial injury^a^	11 353 (81)	1172 (99)
Relative change in hs-cTnT concentrations, median (IQR), %	22 (10-56)	255 (53-1611)
Presence of acute myocardial injury^b^	4396 (31)	991 (84)
First hemoglobin, median (IQR), g/dL	13.0 (11.4-14.5)	13.3 (11.7-14.6)
eGFR at presentation, mL/min/1.73 m^2^^c^		
≥60	4420 (31)	378 (32)
30-59	5538 (39)	529 (45)
<30	4079 (29)	267 (23)
No. of SCr measurements, median (IQR)	4 (3-6)	4 (3-7)
KDIGO stage		
1	9941 (71)	869 (74)
2	2126 (15)	145 (12)
3	1970 (14)	160 (14)
Prior medications		
Aspirin	4595 (33)	526 (45)
P2Y12 inhibitors^d^	782 (6)	107 (9)
Any platelet inhibitor^e^	4981 (35)	561 (48)
β-Blockers	6894 (49)	591 (50)
ACE inhibitor or ARB	6735 (48)	617 (53)
Statins	4577 (33)	470 (40)
Diuretics	2082 (15)	158 (13)
OAC^f^	2488 (18)	121 (10)

Abbreviations: ACE, angiotensin-converting enzyme; ARB, angiotensin receptor blocker; COPD, chronic obstructive pulmonary disease; eGFR, estimated glomerular filtration rate; hs-cTnT, high-sensitivity cardiac troponin T; KDIGO, Kidney Disease Improving Global Outcomes; MI, myocardial infarction; OAC, oral anticoagulant; SCr, serum creatinine.

SI conversion factors: To convert hs-cTnT to micrograms per liter, multiply by 0.001; hemoglobin to grams per liter, multiply by 10.0.

^a^ Any hs-cTnT concentration >14 ng/L.

^b^ Myocardial injury and >20% relative change in hs-cTnT concentrations.

^c^ Calculated according to the Chronic Kidney Disease Epidemiology Collaboration equation.

^d^ Includes treatment with clopidogrel, tikagrelor, dipyradimol, or prasugel.

^e^ Includes treatment with aspirin or P2Y12 inhibitors.

^f^ Includes treatment with any direct oral anticoagulant or warfarin.

Overall, 299 visits (2%) were included on the sole basis of *ICD-10* codes (eTable 2 in Supplement 1). The proportion of patients with an eGFR less than 30 mL/min/1.73 m^2^ at presentation was higher in this group compared with the rest of the study population, and these patients had a slightly higher prevalence of cardiovascular comorbidities. Patients with a diagnosis of MI were more likely than other patients with myocardial injury to undergo both coronary angiography (56% [658 at 1172 visits] vs 3% [386 at 11 406 visits]) and revascularization during the visit (47% [545 at 1172 visits] vs 1% [109 at 11 406 visits]) and to receive any new cardiovascular medication within 30 days (76% [895 at 1172 visits] vs 62% [7085 at 11 406 visits]) (eTable 3 in Supplement 1).

### Prevalence of Myocardial Injury

**Figure 1.**
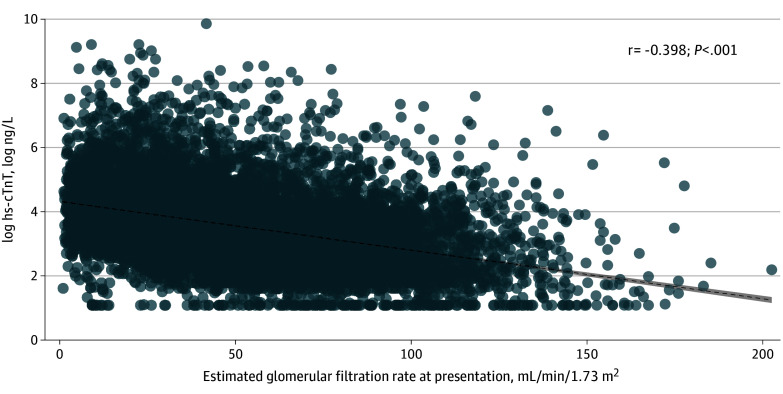
Correlation Between Estimated Glomerular Filtration Rate and log High-Sensitivity Cardiac Troponin T (hs-cTnT) Concentrations at Presentation in Visits by Patients Without Myocardial Infarction To convert hs-cTnT to micrograms per liter, multiply by 0.001.

First hs-cTnT concentrations in patients without MI correlated inversely with eGFR at presentation (correlation coefficient, *r* = −0.40; *P* < .001) (Figure 1). Altogether, 1126 of 1172 visits (96%) by patients with MI and 8937 of 14 037 visits (64%) by patients without MI had 2 or more hs-cTnT concentrations measured during the visit. Both the absolute hs-cTnT concentration and the change in hs-cTnT concentration were higher in patients with MI (Table 1; eFigure 4A and B in Supplement 1). Most patients without an MI (11 353 patients at 14 037 visits [81%]) had evidence of myocardial injury, and almost 1 of 3 patients without an MI (4396 patients at 14 037 visits [31%]) had evidence of acute myocardial injury (Table 1). Both these entities were frequently observed among patients with the most common discharge diagnoses (eFigure 3A-D in Supplement 1). Overall, a larger change in hs-cTnT was observed within higher quartiles of change in SCr (eTable 4 in Supplement 1).

### Dynamic Change in hs-cTnT Concentrations

The estimated change in hs-cTnT concentration increased in a graded manner with the increasing change in SCr concentration in visits by patients without MI, and was 1.8-fold higher in the highest compared with the lowest quartile of change in SCr in adjusted models (64.7% [95% CI, 58.4%-71.5%] vs 36.3% [95% CI, 32.4%-40.7%]; exp(β), 1.78 [95% CI, 1.62-1.96]) (Table 2). In parallel, the prevalence of change in hs-cTnT concentration indicative of acute myocardial injury was 23% (817 of 3507 visits) in the first (lowest) quartile of change in SCr, 29% (1016 of 3521 visits) in the second quartile, 34% (1185 of 3493 visits) in the third quartile, and 39% (1378 of 3516 visits) in the fourth (highest) quartile (Table 3). The corresponding adjusted risks (ORs) of acute myocardial injury were 1.4-fold (OR, 1.37 [95% CI, 1.23-1.53) in the second quartile of change in Scr, 1.8-fold (OR, 1.77 [95% CI, 1.59-1.98]) in the third quartile, and 2.3-fold (OR, 2.32 [95% CI, 2.08-2.59]) in the fourth quartile. (Table 3; eFigure 5 in Supplement 1). Results were similar in analyses in which time between hs-cTnT measurements to define acute myocardial injury was restricted to 12 hours, although associations were slightly attenuated (eTable 5 in Supplement 1).

**Table 2.** Log-Linear Regression Estimate of Relative Change of hs-cTnT in Patients With Acute Kidney Injury and No Diagnosis of Myocardial Infarction^a^

Relative change in SCr levels	Unadjusted model		Multivariable adjusted model^b^	
	exp(β) (95% CI)	Expected relative Δhs-cTnT (95% CI), %^c^	exp(β) (95% CI)	Expected relative Δhs-cTnT (95% CI), %^c^
First quartile	1.00 [Reference]	17.9 (16.8-19.1)	1.00 [Reference]	36.3 (32.4-40.7)
Second quartile	1.46 (1.33-1.61)	26.2 (24.5-28.0)	1.23 (1.12-1.35)	44.5 (40.1-49.4)
Third quartile	1.59 (1.45-1.75)	28.5 (26.7-30.5)	1.37 (1.25-1.51)	49.9 (45.0-55.4)
Fourth quartile	2.09 (1.91-2.29)	37.5 (35.1-40.0)	1.78 (1.62-1.96)	64.7 (58.4-71.5)

Abbreviations: Δhs-cTnT, change in high-sensitivity cardiac troponin T; exp(β), exponentiated β coefficient; hs-cTnT, high-sensitivity cardiac troponin T; SCr, serum-creatinine.

^a^ Patients with very high hs-cTnT concentrations (hs-cTnT >1000 ng/L [to convert to micrograms per liter, multiply by 0.001]) were excluded from the analyses. *P* < .001 for all estimates. There were 8937 of 14 037 visits (64%) with 2 or more hs-cTnT concentrations measured.

^b^ Multivariable adjustment was made for the following variables: age, sex, estimated glomerular filtration rate at presentation, prior myocardial infarction, heart failure, prior stroke, prior chronic obstructive pulmonary disease, atrial fibrillation, and diabetes.

^c^ Estimated by fitting log-linear models with no intercept.

**Table 3.** Risk of Acute Myocardial Injury According to Dynamic Change in Kidney Function in Patients With Acute Kidney Injury and No Diagnosis of Myocardial Infarction

Characteristic	Relative change in SCr levels, No. (%)			
	First quartile	Second quartile	Third quartile	Fourth quartile
All visits				
No. of visits	3507	3521	3493	3516
Visits with acute myocardial injury	817 (23)	1016 (29)	1185 (34)	1378 (39)
Unadjusted OR (95% CI)	1.00 [Reference]	1.34 (1.20-1.49)	1.69 (1.52-1.88)	2.12 (1.91-2.36)
Multivariable adjusted OR (95% CI)^a^	1.00 [Reference]	1.37 (1.23-1.53)	1.77 (1.59-1.98)	2.32 (2.08-2.59)
Visits with ≥2 hs-cTnT measurements				
No. of visits	2232	2231	2237	2237
Visits with acute myocardial injury	856 (38)	1038 (47)	1175 (53)	1327 (59)
Unadjusted OR (95% CI)	1.00 [Reference]	1.40 (1.24-1.58)	1.78 (1.58-2.01)	2.34 (2.07-2.65)
Multivariable adjusted OR (95% CI)^a^	1.00 [Reference]	1.32 (1.16-1.49)	1.71 (1.51-1.94)	2.31 (2.03-2.62)

Abbreviations: hs-cTnT, high-sensitivity cardiac troponin T; OR, odds ratio; SCr, serum-creatinine concentrations.

^a^ Multivariable adjustment was made for the following variables: age, sex, estimated glomerular filtration rate at presentation, prior myocardial infarction, heart failure, prior stroke, prior chronic obstructive pulmonary disease, atrial fibrillation, and diabetes.

The estimated change in hs-cTnT velocity in association with the change in SCr velocity in visits by patients with no MI is illustrated in eFigure 6A and B in Supplement 1. The estimated changes in hs-cTnT velocity were 3.9% (95% CI, 3.5%-4.5%) per hour for an SCr increase of 5% and 4.6% (95% CI, 4.2%-5.0%) per hour for an SCr decrease of 5% (eTable 6 in Supplement 1).

### High-Sensitivity Cardiac Troponin Measurements for Diagnosis of MI

**Figure 2.**
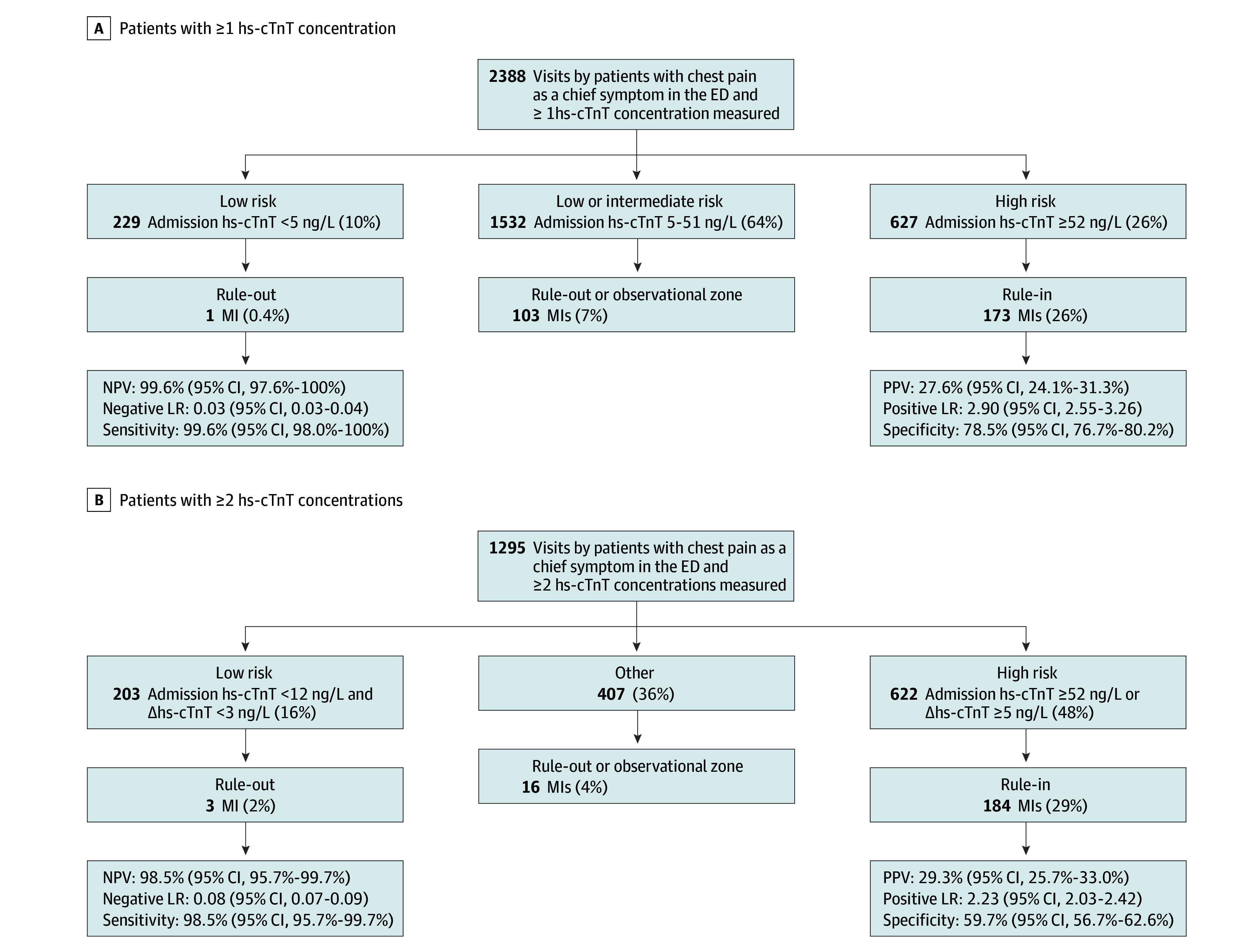
Diagnostic Performance of the European Society of Cardiology Algorithm for Risk Stratification of Myocardial Infarction (MI) in Patients With Chest Pain A, Visits with 1 or more high-sensitivity cardiac troponin t (hs-cTnT) concentration measured. B, Visits with 2 or more hs-cTnT concentrations measured. Δhs-cTnT indicates change in high-sensitivity cardiac troponin T; ED, emergency department; LR, likelihood ratio; NPV, negative predictive value; and PPV, positive predictive value. To convert hs-cTnT to micrograms per liter, multiply by 0.001.

In the subgroup of patients with chest pain as the primary symptom (n = 2388), the clinical accuracy for an MI as quantified by the area under the curve (AUC) for hs-cTnT at admission was 0.78 (95% CI, 0.75-0.81) (eFigure 7 in Supplement 1), and application of the ESC admission hs-cTnT rule-in cutoff criterion of 52 ng/L or more resulted in a positive predictive value (PPV) of 27.6% (95% CI, 24.1%-31.3%), sensitivity of 62.5% (95% CI, 56.8%-68.2%), specificity of 78.5% (95% CI, 76.7%-80.2%), and positive likelihood ratio (LR) of 2.90 (95% CI, 2.55-3.26) (Figure 2; eTable 7 in Supplement 1). A comparable performance was observed with a cutoff value according to the Youdens J-index of 48 ng/L (PPV, 26.8% [95% CI, 23.5-30.1]; sensitivity, 65.7% [95% CI, 60.1%-71.3%]; specificity, 76.4% [95% CI, 74.7%-78.3%]; and positive LR, 2.79 [95% CI, 2.47-3.11]) (eTable 7 in Supplement 1).

A total of 1295 of 2388 patients (54%) had a second hs-cTnT measurement analyzed; in these patients, the performance for diagnosing an MI was slightly higher for the absolute change in hs-cTnT compared with the relative change in hs-cTnT (AUC, 0.85 [95% CI, 0.82-0.88] vs 0.75 [95% CI, 0.70-0.79]) (eFigure 7 in Supplement 1). The ESC rule-in criterion of an absolute change in hs-cTnT of 5 ng/L resulted in a PPV of 32.2% (95% CI, 28.2%-36.3%), specificity of 66.6% (95% CI, 63.7%-69.4%), sensitivity of 85.2% (95% CI, 79.6%-89.8%), and positive LR of 2.55 (95% CI, 2.29-2.81) (eTable 7 in Supplement 1). In comparison, a cut point based on the Youden J-index of 8 ng/L yielded a higher specificity (80.1% [95% CI, 77.6%-82.5%]) and PPV (42.4% [95% CI, 37.4%-47.6%]), but a slightly lower sensitivity (78.8% [95% CI, 72.6%-84.2%]). The correspondingly derived cut point for the relative change in hs-cTnT matched the acute myocardial injury criterion closely (23% vs 20%) and exhibited a similar diagnostic performance. To achieve a specificity of 90% or more, the cutoff level for hs-cTnT at admission was set at 90 ng/mL, for absolute change in hs-cTnT was set at 20 ng/mL, and for relative change in hs-cTnT was set at and 54%; however, these cut points were associated with low sensitivity.

### Diagnostic Performance of hs-cTnT–Based 0-/1-Hour MI Risk Stratification Algorithm Criteria

The ESC rule-in cutoff criterion of hs-cTnT at admission of 52 ng/L or more identified 627 of 2388 patients (26%) at high risk, in whom 173 (28%) MIs occurred (Figure 2A; eTable 7 in Supplement 1). The criterion of hs-cTnT at admission assigned only 229 of 2388 patients (10%) to low risk, while most patients (64% [1532 of 2388]) were triaged toward low or intermediate risk, in whom 103 MIs (7%) occurred (Figure 2A). Findings were similar in patients with 2 or more hs-cTnT measurements, with overall poor ability to identify patients at high risk (Figure 2B).

## Discussion

In this large observational cohort study, we explored the clinical implications of hs-cTnT measurements in visits by patients with AKI in the ED. We found that biochemical evidence of myocardial injury was evident in 4 of 5 visits made by patients without an MI diagnosis, and almost 1 of 3 visits were made by patients with dynamic changes of hs-cTnT indicative of acute myocardial injury. Altogether, more than 80% of all visits with such biochemical evidence of acute myocardial injury were not accompanied with an MI diagnosis. The prevalence of myocardial injury in the ED varies depending on the study setting and is reported mostly from cohorts of patients with symptoms of a suspected MI, with numbers typically ranging from 10% to 35%.^26,27,28,29^ In addition, the proportion of patients with hs-cTnT elevations classified as indicative of acute nonischemic myocardial injury differs substantially between studies.^30^

High-sensitivity cardiac troponin elevations associated with both underlying structural cardiac and kidney disease are commonly observed in patients with CKD and are strongly associated with increased risk of future cardiovascular events and mortality.^8,9,31,32,33,34^ The exact cause of elevated cTnT in patients with decreased kidney function is controversial, partly because the intact troponin molecule is too large to be filtered across the glomerular membrane.^12,35,36^ In settings with high cTnT concentrations—for example, in acute MI—observations suggest a principal extrarenal clearance mechanism by receptor-mediated endocytosis in the liver.^37^ By contrast, prior studies indicate a higher degree of cTnT degradation into fragments small enough for glomerular filtration (<20 kDa) in kidney insufficiency, which would imply a greater importance of kidney cTnT clearance at low and stable cTnT.^35^ Several plausible mechanisms may be responsible for elevated and dynamic cTnT in AKI, including those leading to acute ischemic myocardial injury in conditions with myocardial oxygen supply and demand mismatch indicative of a type 2 MI, and those related to conditions associated with acute nonischemic myocardial injury.^4,38^ The effect of reduced kidney cTnT clearance in AKI is so far unexplored, but probably depends on the AKI phenotype and the clinical setting.

The prevalence of hs-cTnT fluctuations indicative of acute myocardial injury in our study was closely linked to concurrent dynamic changes in SCr. The adjusted risk of having biochemical evidence of acute myocardial injury in patients without an MI diagnosis was more than 2-fold in patients with the highest change in SCr quartile, among whom the change in hs-cTnT concentrations were 1.8 times higher compared with patients with the lowest change in SCr quartile. Furthermore, the estimated changes in hs-cTnT velocity based on changes in SCr per hour were approximately 4% for an SCr increase or decrease of 5%.

Comparisons of results from our study with others modeling change in hs-cTnT velocity on change in SCr are difficult due to differences in study and analysis design.^13,14,15,16,17^ Whether our models could be used to facilitate the interpretation of hs-cTnT concentrations in patients with AKI needs to be further evaluated. Future research should focus on underlying plausible mechanistic concepts, which subsequently would clarify the magnitude of cTnT elevation and kinetic change that could be explained by changes in kidney function in association with concurrent AKI-induced ischemic or nonischemic cardiomyocyte injury. Such clarification would ultimately help to improve cardiovascular risk stratification and preventive treatment strategies.

In subgroup analyses, we found that the performance of the hs-cTnT–based ESC 0-/1-hour MI risk stratification algorithm cutoff values for identifying patients with chest pain at a high risk of MI was poor, with a PPV of less than 30% and concomitant specificities of less than 80% and less than 60% according to either cutoff criteria. The observed reduced clinical accuracy in this population is consistent with findings from other populations of patients with CKD, and is probably associated with a combination of chronically increased hs-cTnT concentrations, multimorbidity, and a high baseline risk of MI.^39,40,41^ Similar to findings from prior studies on early triage of patients with CKD with suspected MI, a large number of patients would be characterized as warranting further observation according to any of these risk stratification criteria, although a minority would have a final MI diagnosis.^39,40^ Safety for patients with AKI assigned to the low-risk group was high, but only 10% had undetectable hs-cTnT concentrations at presentation and thus would have been eligible for early rule-out of MI.

Our findings demonstrate the diagnostic challenges associated with AKI and raise concerns about novel approaches to increase specificity for MI diagnosis in this patient segment. One such approach could be SCr–adjusted hs-cTnT cutoff levels, which has been suggested for patients with CKD.^41^ Novel immunoassays targeting specific epitopes of cTnT fragments may help to differentiate dynamic cTnT elevations explained mainly by nonischemic from ischemic factors according to potentially different cTnT fragmentation patterns during degradation.^33,36,42^ However, the clinical utility of such novel assays in AKI remains unknown.

### Strengths and Limitations

This study has some strengths. The size of the study population allowed estimation of risk estimates with high precision in all analyses. The national Swedish registries used in this study have been thoroughly validated.^19,20,21^ The inclusion of patients from different hospital sizes and locations in Sweden increases the generalizability of the study findings.

This study also has some limitations. Urine output data were not available. Therefore, we could have missed some cases of AKI according to the KDIGO definition.^6^ We were not able to adjudicate the MI diagnoses; thus, the number of potentially missed MIs among patients with acute myocardial injury remains unknown. Specifically, a considerable proportion of patients without a registered MI diagnosis may have met criteria for a type 2 MI.^30,43^ The changes in SCr levels were categorized to account for the skewed distribution of data and to make results interpretable and comprehensible. However, this approach may have inherently obfuscated meaningful association patterns, for example, due to assumption of homogeneity within each category. We used data collected from clinical settings; thus, analyses of hs-cTnT concentrations and SCr were not performed according to a prespecified protocol. Consequently, both selection and ascertainment bias likely affected our study findings.

## Conclusions

In this cohort study, we found that elevated and dynamic hs-cTnT concentrations indicative of acute myocardial injury are common among patients with AKI in the ED and are associated with dynamic changes in SCr. This finding was reflected by a low clinical accuracy of guideline-recommended hs-cTnT cutoff criteria for MI in patients presenting with chest pain to the ED with concurrent AKI.

## Notes

Supplement 1. **eTable 1.** Definition and Staging of Acute Kidney Injury **eTable 2.** Baseline Characteristics in Visits by Patients Included on the Sole Base of ICD-Codes for Acute Kidney Injury (N17) Compared to the Rest of the Study Population **eTable 3.** Management in Patients With Myocardial Injury With and Without a Diagnosis of Myocardial Infarction **eTable 4.** Patient Characteristics Stratified by Dynamic Change in S-Creatinine Concentrations in Patients Without Myocardial Infarction **eTable 5.** Risk of Acute Myocardial Injury in Relation to Dynamic Change in Renal Function With Time Between Blood Samples for hs-cTnT Concentrations to Define Acute Dynamic Change Restricted to 12 Hours **eTable 6.** Predicted hs-cTnT Velocity According to Serum Creatinine Velocity in Patients With and Without Myocardial Infarction **eTable 7.** Diagnostic Performance of hs-cTnT Concentrations for Myocardial Infarction in Patients With Chest Pain **eFigure 1.** Selection of the Study Population **eFigure 2.** Selection of a Subgroup of Patients With Chest Pain at Presentation **eFigure 3.** Prevalence of Myocardial Injury in the Most Common Discharge Diagnoses **eFigure 4.** Kernel Density Plots of hs-cTnT Concentrations in Patients With and Without Myocardial Infarction **eFigure 5.** Adjusted Odds Ratios for Acute Myocardial Injury in Visits by Patients Without Myocardial Infarction **eFigure 6.** Estimated Hs-cTnT Velocity in Relation to Serum Creatinine Velocity **eFigure 7.** Receiver Operating Characteristic Curves (ROCs) of hs-cTnT Measures for Diagnosing Myocardial Infarction

Supplement 2.
Data Sharing Statement
