# Supplementary material for: Acute Kidney Injury and High-Sensitivity Cardiac Troponin T Levels in the Emergency Department
Source: JAMA Netw Open. 2024 Aug 30;7(8):e2419602. doi: 10.1001/jamanetworkopen.2024.19602 (PMC11365000; doi:10.1001/jamanetworkopen.2024.19602)
Supplement: Supplement 1. — eTable 1. Definition and Staging of Acute Kidney Injury eTable 2. Baseline Characteristics in Visits by Patients Included on the Sole Base of ICD-Codes for Acute Kidney Injury (N17) Compared to the Rest of the Study Population eTable 3. Management in Patients With Myocardial Injury With and Without a Diagnosis of Myocardial Infarction eTable 4. Patient Characteristics Stratified by Dynamic Change in S-Creatinine Concentrations in Patients Without Myocardial Infarction eTable 5. Risk of Acute Myocardial Injury in Relation to Dynamic Change in Renal Function With Time Between Blood Samples for hs-cTnT Concentrations to Define Acute Dynamic Change Restricted to 12 Hours eTable 6. Predicted hs-cTnT Velocity According to Serum Creatinine Velocity in Patients With and Without Myocardial Infarction eTable 7. Diagnostic Performance of hs-cTnT Concentrations for Myocardial Infarction in Patients With Chest Pain eFigure 1. Selection of the Study Population eFigure 2. Selection of a Subgroup of Patients With Chest Pain at Presentation eFigure 3. Prevalence of Myocardial Injury in the Most Common Discharge Diagnoses eFigure 4. Kernel Density Plots of hs-cTnT Concentrations in Patients With and Without Myocardial Infarction eFigure 5. Adjusted Odds Ratios for Acute Myocardial Injury in Visits by Patients Without Myocardial Infarction eFigure 6. Estimated Hs-cTnT Velocity in Relation to Serum Creatinine Velocity eFigure 7. Receiver Operating Characteristic Curves (ROCs) of hs-cTnT Measures for Diagnosing Myocardial Infarction [file jamanetwopen-e2419602-s001.pdf]

Supplementary Online Content

Cyon L, Kadesjö E, Edgren G, Roos A. Acute kidney injury and high-sensitivity cardiac troponin T levels in the emergency department. *JAMA Netw Open.* 2024;7(8):e2419602. doi:10.1001/jamanetworkopen.2024.19602

eTable 1. Definition and Staging of Acute Kidney Injury
eTable 2. Baseline Characteristics in Visits by Patients Included on the Sole Base of ICD-Codes for Acute Kidney Injury (N17) Compared to the Rest of the Study Population
eTable 3. Management in Patients With Myocardial Injury With and Without a Diagnosis of Myocardial Infarction
eTable 4. Patient Characteristics Stratified by Dynamic Change in S-Creatinine Concentrations in Patients Without Myocardial Infarction
eTable 5. Risk of Acute Myocardial Injury in Relation to Dynamic Change in Renal Function With Time Between Blood Samples for hs-cTnT Concentrations to Define Acute Dynamic Change Restricted to 12 Hours
eTable 6. Predicted hs-cTnT Velocity According to Serum Creatinine Velocity in Patients With and Without Myocardial Infarction
eTable 7. Diagnostic Performance of hs-cTnT Concentrations for Myocardial Infarction in Patients With Chest Pain
eFigure 1. Selection of the Study Population
eFigure 2. Selection of a Subgroup of Patients With Chest Pain at Presentation
eFigure 3. Prevalence of Myocardial Injury in the Most Common Discharge Diagnoses
eFigure 4. Kernel Density Plots of hs-cTnT Concentrations in Patients With and Without Myocardial Infarction
eFigure 5. Adjusted Odds Ratios for Acute Myocardial Injury in Visits by Patients Without Myocardial Infarction
eFigure 6. Estimated Hs-cTnT Velocity in Relation to Serum Creatinine Velocity
eFigure 7. Receiver Operating Characteristic Curves (ROCs) of hs-cTnT Measures for Diagnosing Myocardial Infarction

This supplementary material has been provided by the authors to give readers additional information about their work.

© 2024 Cyon L et al. *JAMA Network Open.*

eTable 1. Definition and Staging of Acute Kidney Injury

**Definition of AKI**	
**i.**	↑↓S-Cr ≥26.5 µmol/l within 48 hrs during the visit
**ii.**	↑S-Cr to ≥1.5 times the most recent S-Cr within 7 days or<br>↑S-Cr to ≥1.5 times the lowest S-Cr during the visit
**iii.**	↓S-Cr with the highest S-Cr being ≥1.5 times the lowest S-Cr during the visit
**iv.**	ICD 10-code of N17 registered at the time of the visit
**AKI stage**	
**I**. Inclusion criteria according to **i.**	
**II**. A multiplicative S-Cr change of 2.0-2.9 according to inclusion criterion **ii.** or **iii.**	
**III**. A multiplicative S-Cr change of ≥3 S-Cr according to **ii.** or **iii.**, or any ↑S-Cr to ≥354 µmol/l	

Abbreviations: AKI: Acute kidney injury; S-Cr: Serum Creatinine.

© 2024 Cyon L et al. *JAMA Network Open.*

eTable 2. Baseline Characteristics in Visits by Patients Included on the Sole Base of ICD-Codes for Acute Kidney Injury (N17) Compared to the Rest of the Study Population

	**Inclusion only by ICD-codes,** No. (%)	**Others,** No. (%)
Number of visits, (row-%)	299 (2.0)	14,912 (98)
Number of unique patients	277 (93)	13,361 (90)
Age (years), median (IQR)	77 (68-86)	74 (64-83)
**Sex**		
Female	111 (37)	6391 (43)
Male	188 (63)	8521 (57)
**Comorbidities**		
Prior MI	55 (18)	2649 (18)
Prior revascularization	46 (15)	2291 (15)
Prior stroke	64 (21)	2168 (15)
Heart failure	119 (40)	4652 (31)
Diabetes	115 (38)	3887 (26)
Hypertension	206 (69)	8586 (58)
Atrial fibrillation	106 (35)	4334 (29)
COPD	46 (15)	2473 (17)
**Laboratory data**		
First hs-cTnT concentration (ng/l), median (IQR)	53 (29-122)	35 (17-73)
Number of hs-cTnT measurements, median (IQR)	2 (1-3)	3 (3-5)
Number of visits with ≥2 hs-cTnT measured	154 (52)	9909 (66)
Peak hs-cTnT concentration (ng/l), median (IQR)	59 (30-141)	44 (20-108)
Presence of myocardial injury*	276 (92)	12,249 (82)
Relative change of hs-cTnT concentrations (Δhs-cTnT), %, median (IQR)	13 (6-26)	25 (11-80)
Presence of acute myocardial injury†	51 (17)	5336 (36)
First hemoglobin (g/l), median (IQR)	124 (108-141)	130 (115-145)
eGFR at presentation (ml/min/1.73 m2)§:		
>60	20 (6.7)	4778 (32)
30-59	96 (32)	5971 (40)

© 2024 Cyon L et al. *JAMA Network Open.*

<30	183 (61)	4163 (28)
Number of S-Cr measurements, median (IQR)	4 (3-6)	4 (3-7)
**Prior medications**		
Aspirin	112 (37)	5009 (34)
P2Y12 inhibitors‖	23 (7.7)	866 (5.8)
Any platelet inhibitor#	125 (42)	5417 (36)
Beta-blockers	155 (51)	7330 (49)
ACEi/ARB	155 (51)	7197 (48)
Statins	106 (35)	4941 (33)
Diuretics	43 (14)	2197 (15)
OAC**	49 (16)	2560 (10)

*Any hs-cTnT concentration >14 ng/l. †Myocardial injury and >20% relative change in hs-cTnT concentrations concentrations (Δhs-cTnT). §Calculated according to the Chronic Kidney Disease Epidemiology Collaboration equation. ‖Includes treatment with Clopidogrel, Tikagrelor, Dipyradimol or Prasugel. #Includes treatment with Aspirin or P2Y12 inhibitors. **Includes treatment with any DOAC or Warfarin.
Abbreviations: ACEi/ARB: angiotensin-converting enzyme inhibitor/angiotensin receptor blocker; COPD: chronic obstructive pulmonary disease; hs-cTnT: high-sensitivity cardiac troponin T; eGFR: estimated glomerular filtration rate; MI: myocardial infarction; OAC: oral anticoagulants; S-Cr: Serum Creatinine.

© 2024 Cyon L et al. *JAMA Network Open.*

eTable 3. Management in Patients With Myocardial Injury With and Without a Diagnosis of Myocardial Infarction

	**Visits without MI,** No. (%)	**Visits with MI,** No. (%)
Number of visits (row-%)	11,406 (91)	1172 (9.3)
**Coronary intervention**		
Coronary angiography		
Event during the index visit	386 (3.4)	658 (56)
Events conducted after fulfilled AKI-criteria	179 (46)	267 (41)
Events within 30 days	473 (4.2)	682 (58)
Events conducted after fulfilled AKI-criteria	240 (51)	288 (42)
Revascularization		
Event during the index visit	109 (1.0)	545 (47)
Events conducted after fulfilled AKI-criteria	59 (54)	219 (40)
Events within 30 days, n (%)	156 (1.4)	587 (50)
Events conducted after fulfilled AKI-criteria	91 (58)	241 (41)
**New medical treatment**		
Any platelet inhibitor*	3256 (29)	800 (68)
Beta-blockers	5133 (45)	769 (66)
ACEi/ARB	4118 (36)	615 (52)
Statins	2769 (24)	688 (55)
Any cardiovascular medication†	7085 (62)	895 (76)

*Includes any new treatment with Clopidogrel, Tikagrelor, Dipyradimol or Prasugel. †Any new treatment with a platelet inhibitor, statins, betablockers or angiotensin-converting enzyme inhibitor/angiotensin receptor blockers. Abbreviations: ACEi/ARB: angiotensin-converting enzyme inhibitor/angiotensin receptor blocker; AKI: acute kidney injury.

© 2024 Cyon L et al. *JAMA Network Open.*

eTable 4. Patient Characteristics Stratified by Dynamic Change in S-Creatinine Concentrations in Patients Without Myocardial Infarction

	**Relative change in S-Cr (ΔS-Cr),** No. (%)			
	1st quartile	2nd quartile	3rd quartile	4th quartile
Number of visits	3507	3521	3493	3516
Age (years), median (IQR)	76 (67-85)	74 (64-83)	73 (63-83)	73 (62-82)
**Sex**				
Female	1180 (34)	1607 (46)	1679 (48)	1606 (46)
Male	2327 (66)	1914 (54)	1814 (52)	1910 (54)
**Comorbidities**				
Prior MI	736 (31)	567 (24)	522 (22)	550 (23)
Prior revascularization	662 (32)	534 (26)	407 (20)	466 (23)
Prior stroke	566 (16)	540 (15)	506 (14)	468 (13)
Heart failure	1358 (39)	1049 (30)	1059 (30)	1011 (29)
Diabetes	1095 (31)	882 (25)	847 (24)	808 (23)
Hypertension	2309 (66)	1923 (55)	1967 (56)	1864 (53)
Atrial fibrillation	1227 (35)	1028 (29)	998 (29)	949 (27)
COPD	570 (16)	608 (17)	618 (18)	594 (17)
**Laboratory data**				
First hs-cTnT concentration (ng/l), median (IQR)	41 (22-78)	27 (13-52)	32 (16-66)	33 (15-67)
Peak hs-cTnT concentration (ng/l), median (IQR)	45 (23-92)	31 (15-66)	39 (19-84)	44 (19-106)
Presence of myocardial injury*	3046 (87)	2661 (76)	2831 (81)	2815 (25)
Relative change of hs-cTnT concentrations (Δhs-cTnT), %, median (IQR)	16 (7-32)	21 (10-50)	23 (10-54)	34 (12-112)
Presence of acute myocardial injury†	817 (23)	1016 (29)	1185 (34)	1378 (39)
First hemoglobin (g/l), median (IQR)	126 (110-142)	132 (116-146)	132 (116-146)	130 (116-146)
eGFR at presentation (ml/min/1.73 m2)§:				
>60	309 (8.8)	1337 (38)	1231 (35)	1543 (44)
30-59	1621 (46)	1684 (45)	1404 (40)	929 (26)
<30	1577 (45)	600 (17)	858 (25)	1044 (30)
**Prior medications**				
Aspirin	1280 (37)	1138 (32)	1109 (32)	1068 (30)

© 2024 Cyon L et al. *JAMA Network Open.*

P2Y12 inhibitors‖	233 (6.6)	192 (5.5)	182 (5.2)	175 (5.0)
Any platelet inhibitor#	1400 (40)	1237 (35)	1196 (34)	1148 (33)
Beta-blockers	1969 (56)	1677 (48)	1619 (46)	1629 (46)
ACEi/ARB	1934 (55)	1603 (46)	1603 (46)	1595 (45)
Statins	1326 (38)	1139 (32)	1032 (30)	1080 (31)
Diuretics	641 (18)	472 (13)	496 (14)	473 (13)
OAC**	724 (21)	636 (18)	588 (17)	540 (15)

*Any hs-cTnT concentration >14 ng/l. †Myocardial injury and >20% relative change in hs-cTnT concentrations (Δhs-cTnT). §Calculated according to the Chronic Kidney Disease Epidemiology Collaboration equation. ‖Includes treatment with Clopidogrel, Tikagrelor, Dipyradimol or Prasugel. #Includes treatment with Aspirin or P2Y12 inhibitors. **Includes treatment with any DOAC or Warfarin. Abbreviations: ACEi/ARB: angiotensin-converting enzyme inhibitor/angiotensin receptor blocker; COPD: chronic obstructive pulmonary disease; hs-cTnT: high-sensitivity cardiac troponin T; eGFR: estimated glomerular filtration rate; MI: myocardial infarction; OAC: oral anticoagulants; S-Cr: Serum creatinine.

© 2024 Cyon L et al. *JAMA Network Open.*

eTable 5. Risk of Acute Myocardial Injury in Relation to Dynamic Change in Renal Function With Time Between Blood Samples for hs-cTnT Concentrations to Define Acute Dynamic Change Restricted to 12 Hours

	**Relative change in S-Cr levels (ΔS-Cr),** No. (%)			
	1st quartile	2nd quartile	3rd quartile	4th quartile
**All visits**				
Number of visits	3507	3521	3493	3516
Visits with acute myocardial injury	609 (17)	757 (22)	830 (24)	963 (27)
Unadjusted OR, (95% CI)	1.0 (Ref.)	1.30 (1.16-1.47)	1.48 (1.32-1.67)	1.79 (1.60-2.01)
**Multivariable adjusted OR***, (95% CI)	1.0 (Ref.)	**1.28 (1.13-1.45)**	**1.50 (1.33-1.69)**	**1.87 (1.66 -2.12)**
**Visits with ≥ 2 hs-cTnT measurements**				
Number of visits	2232	2231 (25)	2237 (25)	2237 (25)
Visits with acute myocardial injury	637 (29)	769 (34)	821 (37)	920 (41)
Unadjusted OR, (95% CI)	1.0 (Ref.)	1.32 (1.16-1.50)	1.45 (1.28-1.65)	1.75 (1.54-1.98)
**Multivariable adjusted OR***, (95% CI)	1.0 (Ref.)	**1.21 (1.06-1.38)**	**1.36 (1.19-1.55)**	**1.67 (1.46-1.90)**

*Multivariable adjustment was made for the following variables: age, sex, eGFR at presentation, prior myocardial infarction, heart failure, prior stroke, prior chronic obstructive pulmonary disease, atrial fibrillation and diabetes. Abbreviations: CI: confidence interval; hs-cTnT: high-sensitivity cardiac troponin T; OR: odds ratio; S-Cr: Serum-Creatinine concentrations.

© 2024 Cyon L et al. *JAMA Network Open.*

eTable 6. Predicted hs-cTnT Velocity According to Serum Creatinine Velocity in Patients With and Without Myocardial Infarction

	**Predicted hs-cTnT velocity,** Δhs-cTnT per hour (95% CI)	
	Increasing S-Cr	Decreasing S-Cr
**Creatinine velocity**, ΔS-Cr per hour		
5%	3.9% (3.5-4.5)	4.6% (4.2-5.0)
7.5%	5.9% (5.1-6.7)	8.4% (7.6-9.3)
10%	7.9% (6.8-9.1)	13.4% (11.9-15.1)
12.5%	10.0% (8.5-11.8)	19.5% (17.0-22.4)
15%	12.0% (10.1-14.3)	27.5% (23.5-32.2)
20%	16.6% (13.6-20.2)	47.3% (39.1-57.1)

Abbreviations: CI: confidence interval; hs-cTnT: high-sensitivity cardiac troponin T; S-Cr: Serum creatinine.

© 2024 Cyon L et al. *JAMA Network Open.*

eTable 7. Diagnostic Performance of hs-cTnT Concentrations for Myocardial Infarction in Patients With Chest Pain

	Cut-off level	Sensitivity (95% CI)	Specificity (95% CI)	PPV (95% CI)	Ruled-in No. (%)	FP No. (%)	FN No. (%)	NPV (95% CI)	LR+ (95% CI)
**0 h hs-cTnT** (n=2388)									
ESC rule-in criterion	52 ng/l	62.5 (56.8-68.2)	78.5 (76.7-80.2)	27.6 (24.1-31.3)	627 (26)	454 (72)	104 (5.9)	94.1 (92.9-95.2)	2.90 (2.55-3.26)
99th percentile URL	15 ng/l	92.1 (88.9-95.2)	33.0 (31.0-35.0)	15.3 (13.6-17.0)	1669 (70)	1414 (85)	22 (3)	96.9 (95.7-98.2)	1.37 (1.31-1.44)
Youden's J Index	48 ng/l	65.7 (60.1-71.3)	76.4 (74.7-78.3)	26.8 (23.5-30.1)	679 (28)	497 (21)	95 (34)	94.4 (93.4-95.5)	2.79 (2.47-3.11)
Sensitivity optimized cut-off (≥90%)	17 ng/l	90.3 (86.1-93.5)	38.5 (36.4-40.6)	16.1 (14.3-18.1)	1549 (65)	1299 (54)	27 (9.7)	96.7 (95.4-97.9)	1.47 (1.39-1.54)
Specificity optimized cut-off (≥90%)	90 ng/l	43.7 (37.8-49.7)	90.0 (88.7-91.3)	36.5 (31.3-41.9)	332 (14)	211 (64)	156 (56)	92.4 (91.2-93.5)	4.37 (3.56-5.18)
**Absolute Δhs-cTnT** (n=1295)									
ESC rule-in criterion	5 ng/l	85.2 (79.6-89.8)	66.6 (63.7-69.4)	32.2 (28.2-36.3)	538 (42)	365 (68)	30 (4.0)	96.0 (94.4-97.3)	2.55 (2.29 -2.81)
Youden's J Index	8 ng/l	78.8 (72.6-84.2)	80.1 (77.6-82.5)	42.4 (37.4-47.6)	377 (29)	217 (58)	43 (4.7)	95.3 (93.7-96.7)	2.97 (2.42-3.52)
Sensitivity optimized cut-off (≥90%)	3 ng/l	90.0 (84.9-93.4)	54.8 (51.8-57.7)	26.8 (23.5-30.3)	675 (52)	494 (73)	22 (3.5)	96.5 (94.7-97.8)	1.97 (1.81-2.13)
Specificity optimized cut-off (≥90%)	20 ng/l	60.6 (53.5-67.4)	90.4 (88.5-92.1)	54.0 (47.2-60.6)	228 (18)	105 (46)	80 (7.5)	92.5 (90.8-94.0)	6.30 (4.96-7.64)
**Relative Δhs-cTnT** (n=1295)									
Acute myocardial injury criterion	20%	66.5 (59.6-73.0)	74.4 (71.9-77.0)	32.6 (28.1-37.4)	414 (32)	279 (67)	68 (7.7)	92.3 (90.3-94.0)	2.60 (2.24-2.97)
Youden's J Index	23%	63.6 (56.5-70.1)	78.8 (76.3-81.1)	35.7 (30.8-40.7)	361 (28)	232 (64)	74 (8.0)	92.1 (90.2-93.7)	2.99 (2.53 -3.45)

© 2024 Cyon L et al. *JAMA Network Open.*

Sensitivity optimized cut-off (≥90%)	3%	19.8 (17.5-22.3)	90.2 (85.2 -93.9)	17.3 (15.1-19.7)	1059 (82)	876 (83)	20 (8.5)	91.5 (87.2-94.8)	1.12 (1.06-1.18)
Specificity optimized cut-off (≥90%)	54%	48.3 (41.2-55.4)	90.5 (88.6-92.2)	47.6 (40.6-54.6)	206 (16)	108 (52)	105 (9.6)	90.4 (88.5-92.1)	4.88 (3.76-6.00)

Abbreviations: FN; false negatives; hs-cTnT: high-sensitivity cardiac troponin T; LR+: positive likelihood ratio, PPV: positive predictive value; NPV: negative predictive value, URL: upper reference limit.

© 2024 Cyon L et al. *JAMA Network Open.*

eFigure 1. Selection of the Study Population

Abbreviations: AKI: acute kidney injury; hs-cTnT: high-sensitivity cardiac troponin T.

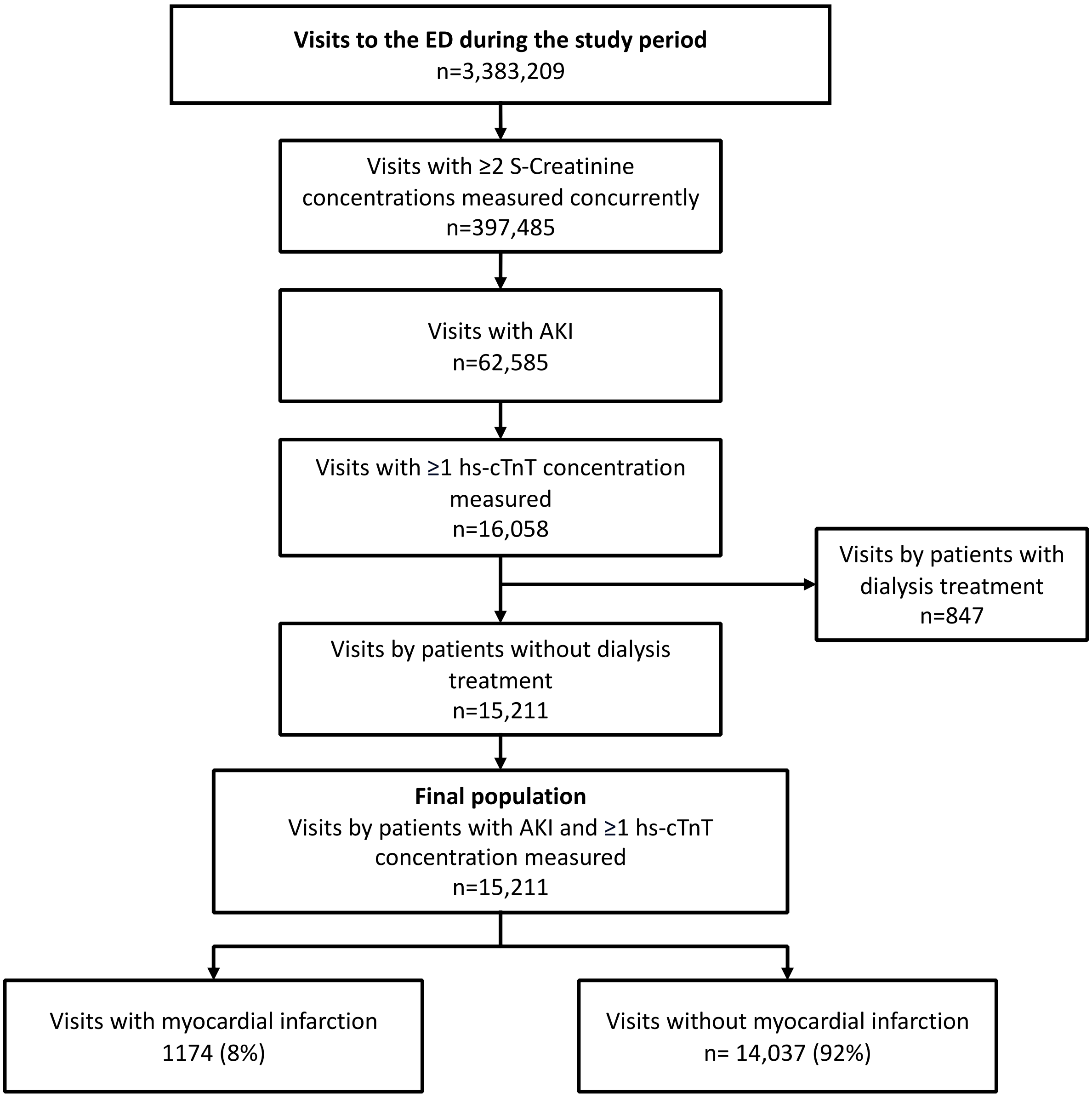

© 2024 Cyon L et al. *JAMA Network Open.*

eFigure 2. Selection of a Subgroup of Patients With Chest Pain at Presentation

Abbreviations: AKI: acute kidney injury; ED: emergency department; hs-cTnT: high-sensitivity cardiac troponin T; STEMI: ST-segment elevation myocardial infarction.

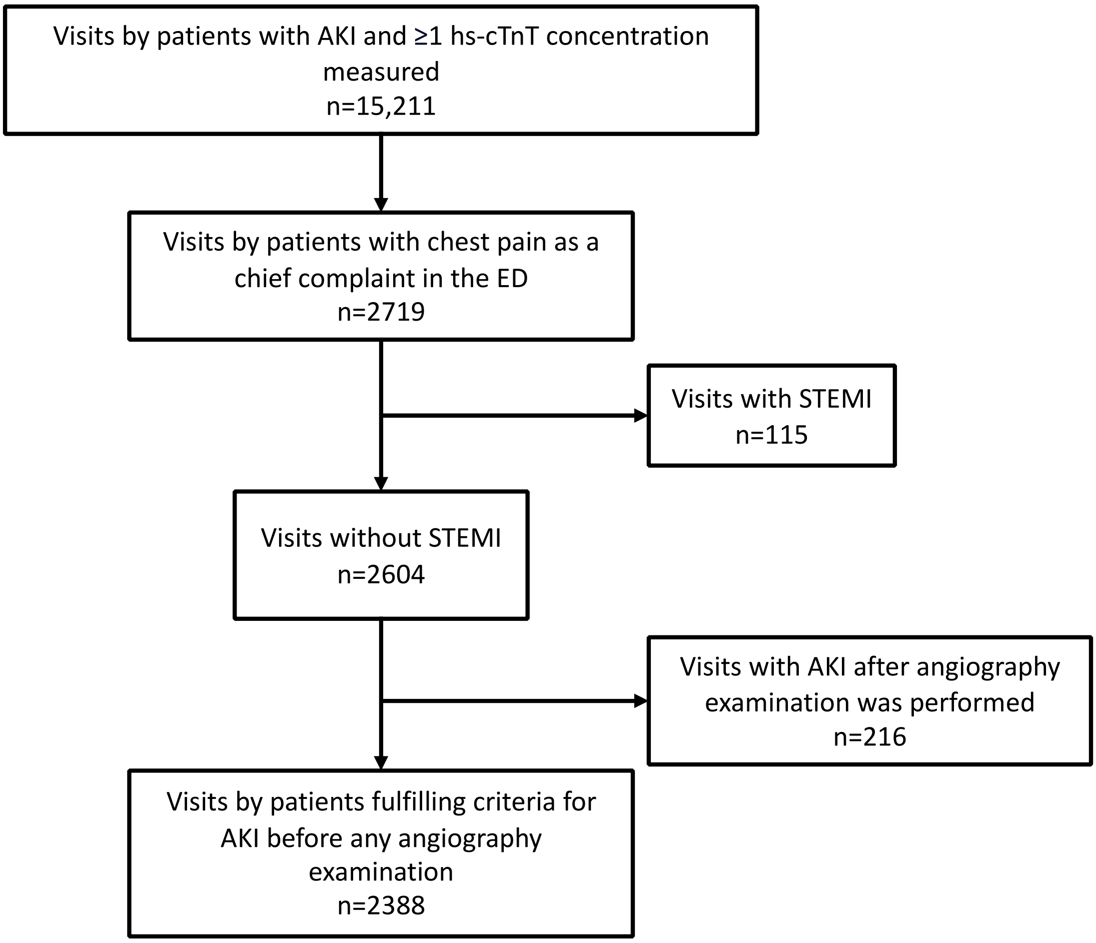

© 2024 Cyon L et al. *JAMA Network Open.*

eFigure 3. Prevalence of Myocardial Injury in the Most Common Discharge Diagnoses
Abbreviations: AKI: acute kidney injury; COPD: chronic obstructive pulmonary disease; PE: pulmonary embolism; TBI: traumatic brain injury; UTI: urinary tract infection. Symptom diagnoses were defined as any ICD-10 code in the R-chapter.

Panel A. Frequency of myocardial injury

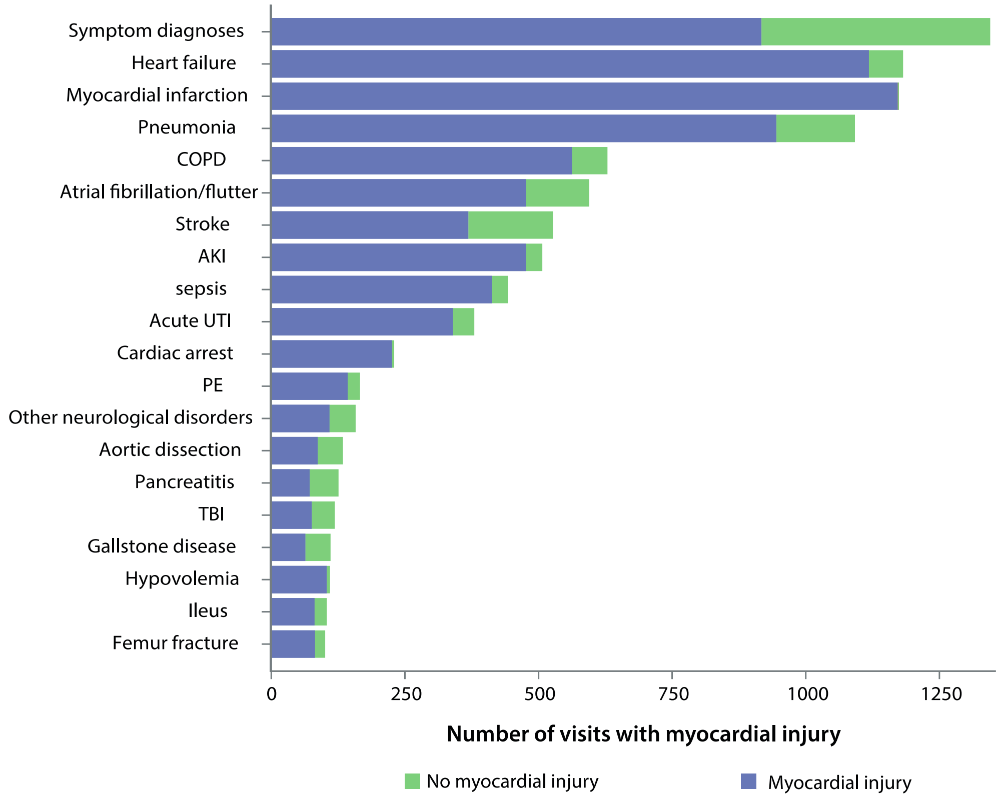

Panel B. Proportion of myocardial injury

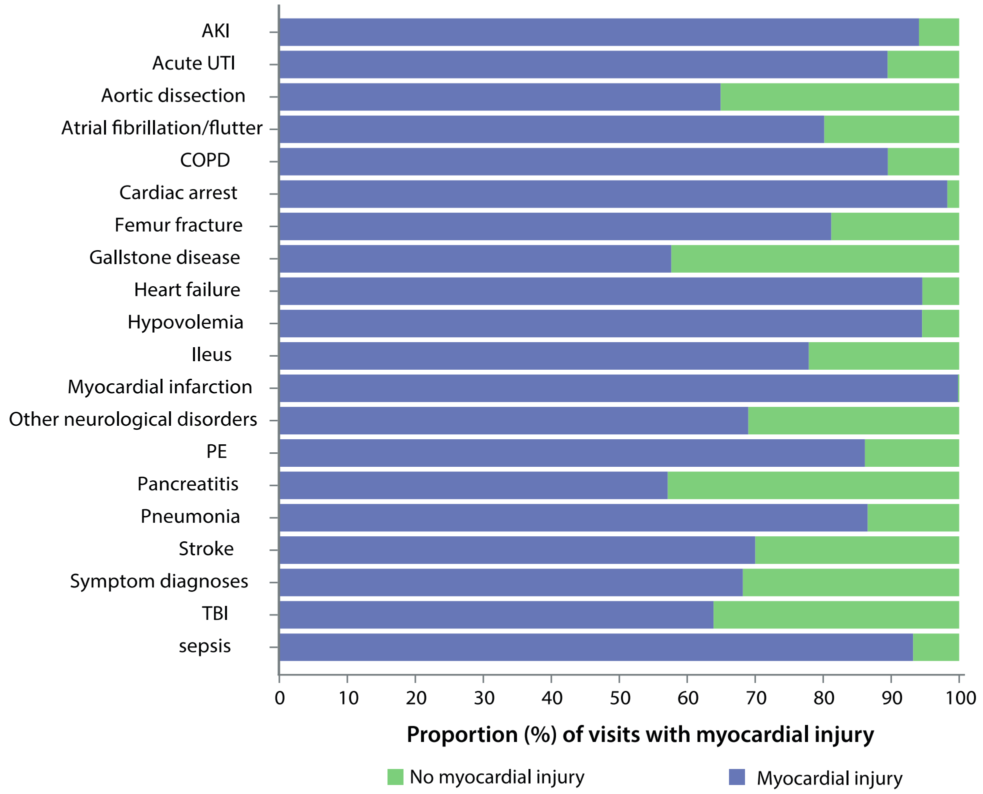

© 2024 Cyon L et al. *JAMA Network Open.*

Panel C. Frequency of acute myocardial injury

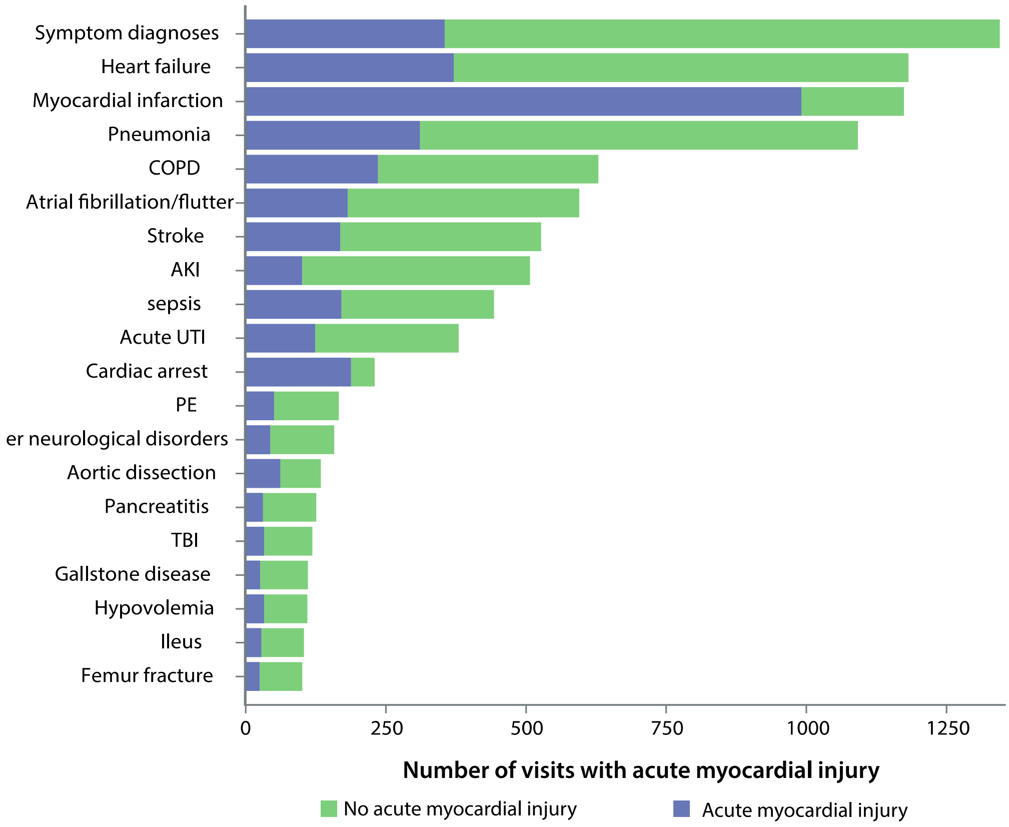

Panel D. Proportion of acute myocardial injury

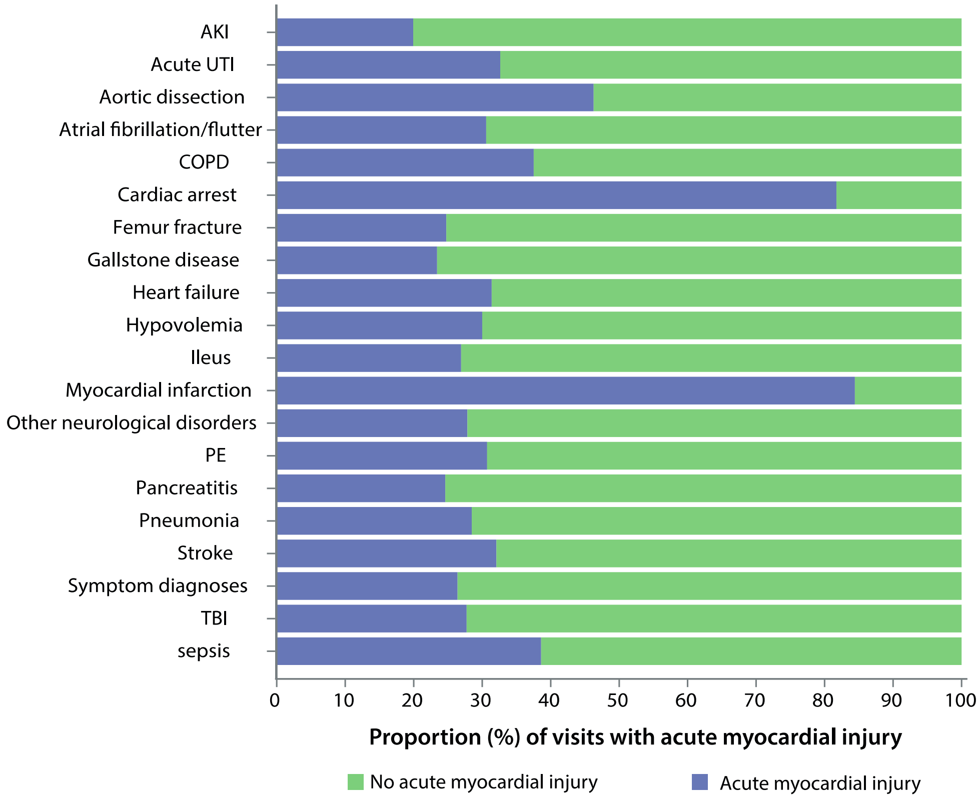

© 2024 Cyon L et al. *JAMA Network Open*.

eFigure 4. Kernel Density Plots of hs-cTnT Concentrations in Patients With and Without Myocardial Infarction

Abbreviations: Hs-cTnT: high-sensitivity cardiac troponin T.

Panel A. Peak Hs-cTnT concentrations

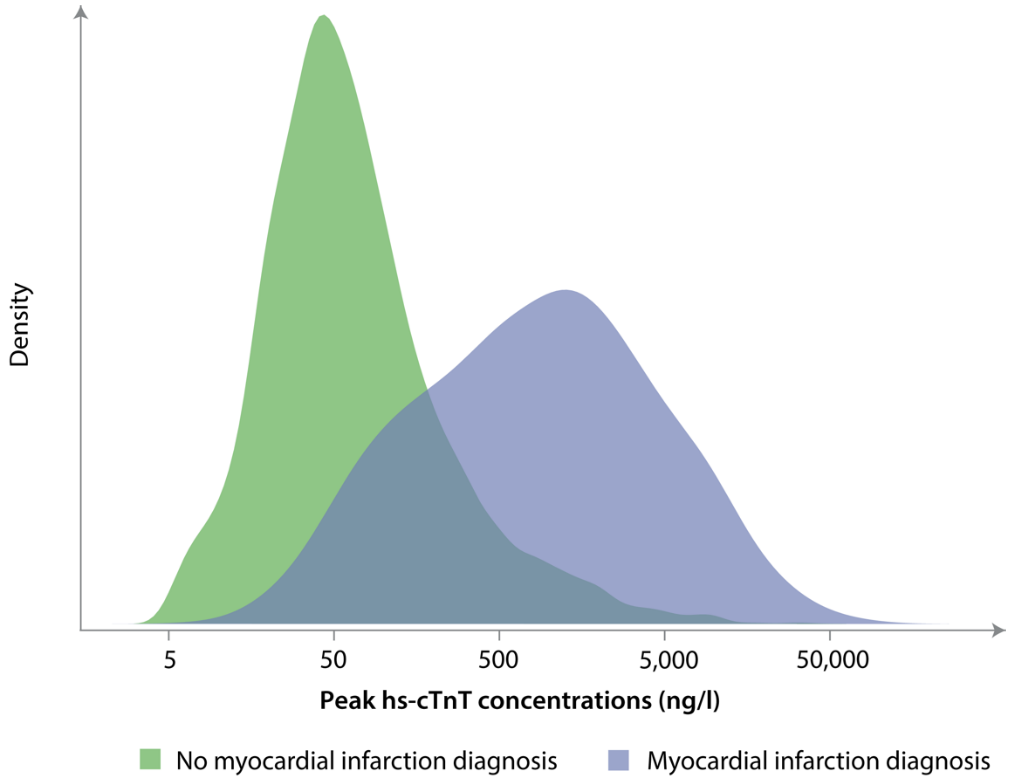

Panel B. Relative change in hs-cTnT concentrations (Δhs-cTnT)

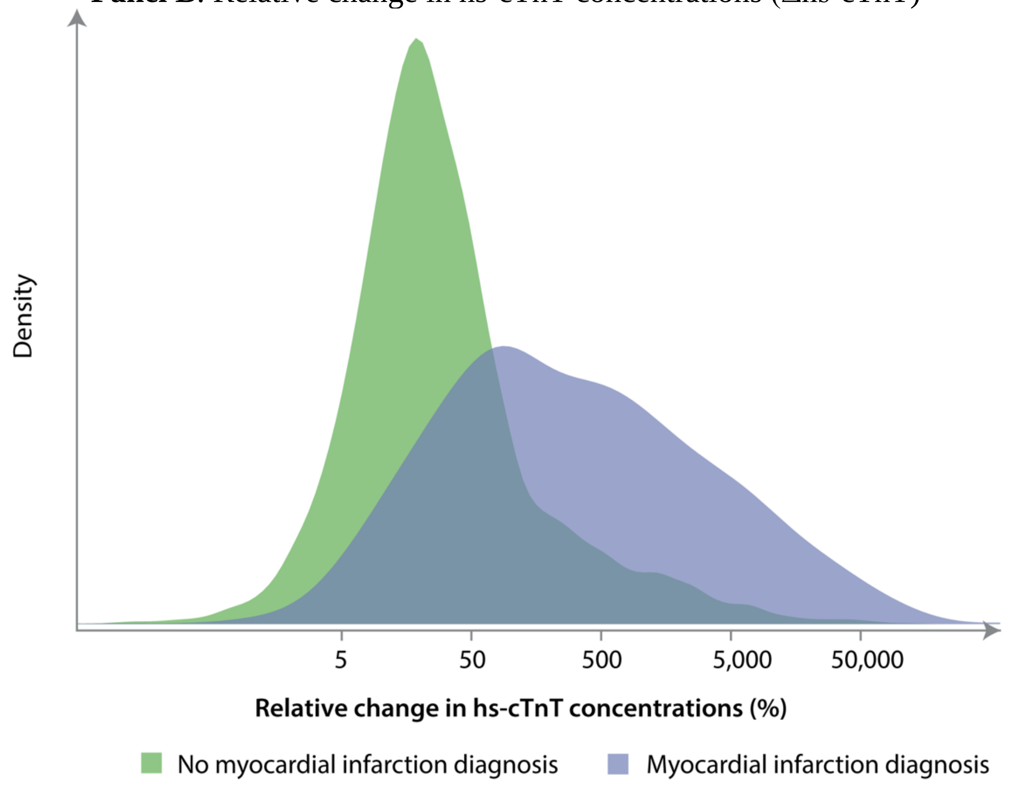

© 2024 Cyon L et al. *JAMA Network Open.*

eFigure 5. Adjusted Odds Ratios for Acute Myocardial Injury in Visits by Patients Without Myocardial Infarction

Abbreviations: AF: atrial fibrillation; aOR: adjusted odds ratio; CI: confidence interval; COPD: chronic obstructive pulmonary disease; DM: diabetes mellitus; eGFR: estimated glomerular filtration rate; HF: heart failure; MI: myocardial infarction.

Panel A. All visits

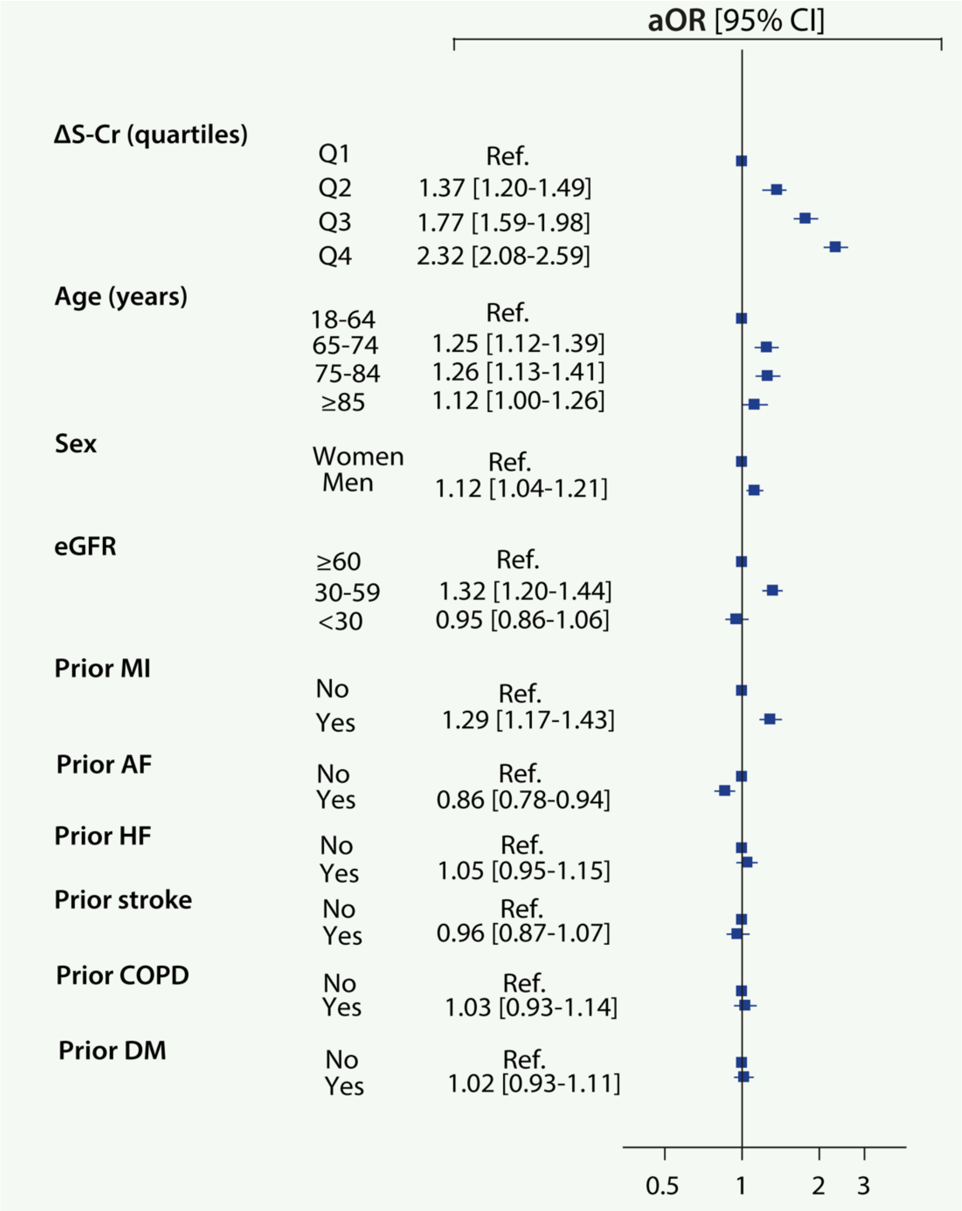

© 2024 Cyon L et al. *JAMA Network Open.*

Panel B. Visits with ≥2 hs-cTnT concentrations measured.

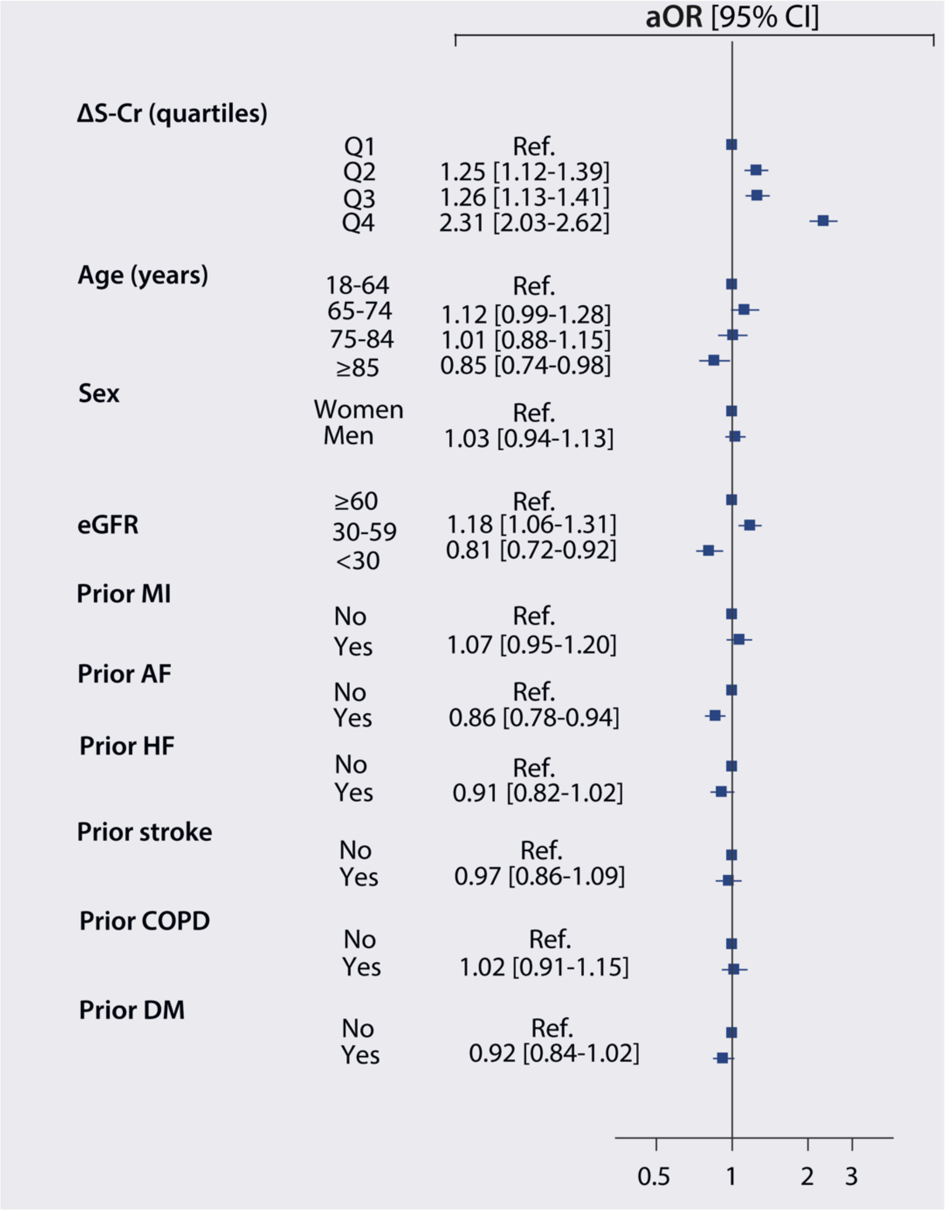

© 2024 Cyon L et al. *JAMA Network Open.*

eFigure 6. Estimated Hs-cTnT Velocity in Relation to Serum Creatinine Velocity
Abbreviations: hs-cTnT: high-sensitivity cardiac troponin T, S-Cr: Serum creatinine.

Panel A. Hs-cTnT velocity with increasing S-Cr concentrations

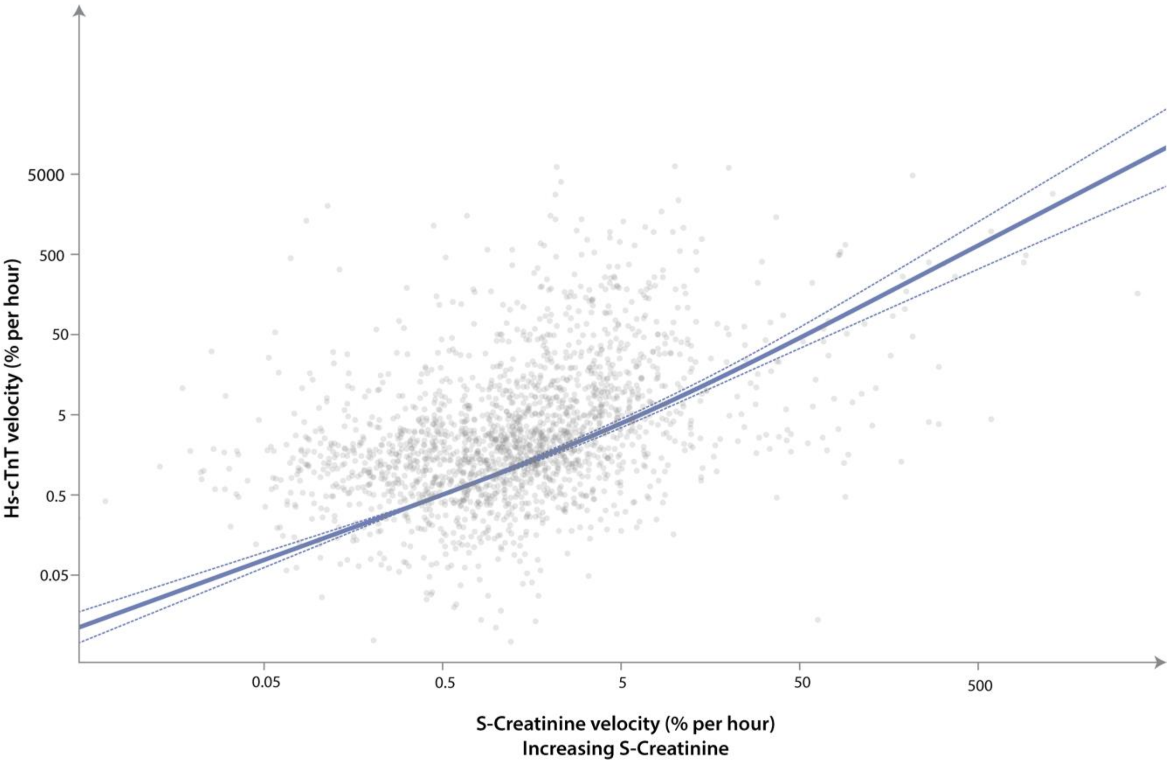

Panel B. Hs-cTnT velocity with decreasing S-Cr concentrations

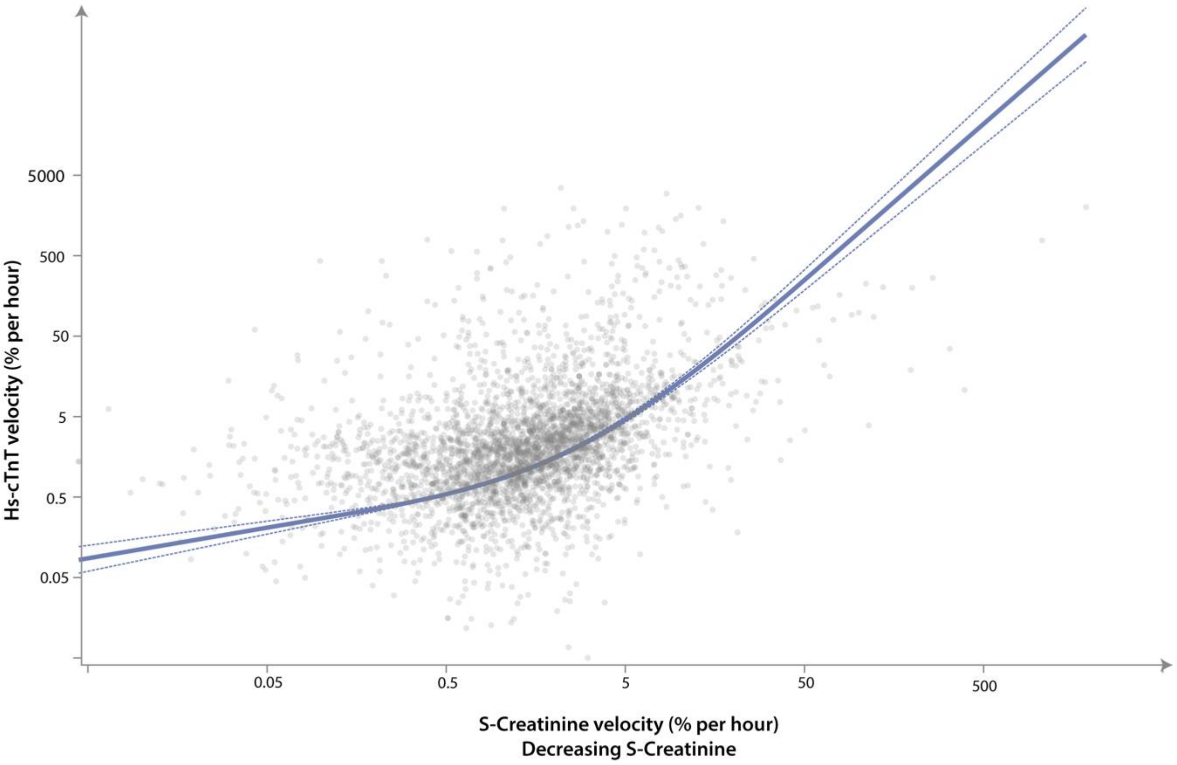

© 2024 Cyon L et al. *JAMA Network Open.*

eFigure 7. Receiver Operating Characteristic Curves (ROCs) of hs-cTnT Measures for Diagnosing Myocardial Infarction

Abbreviations: AUC: area under the curve; CI: confidence interval; hs-cTnT: high-sensitivity cardiac troponin T.

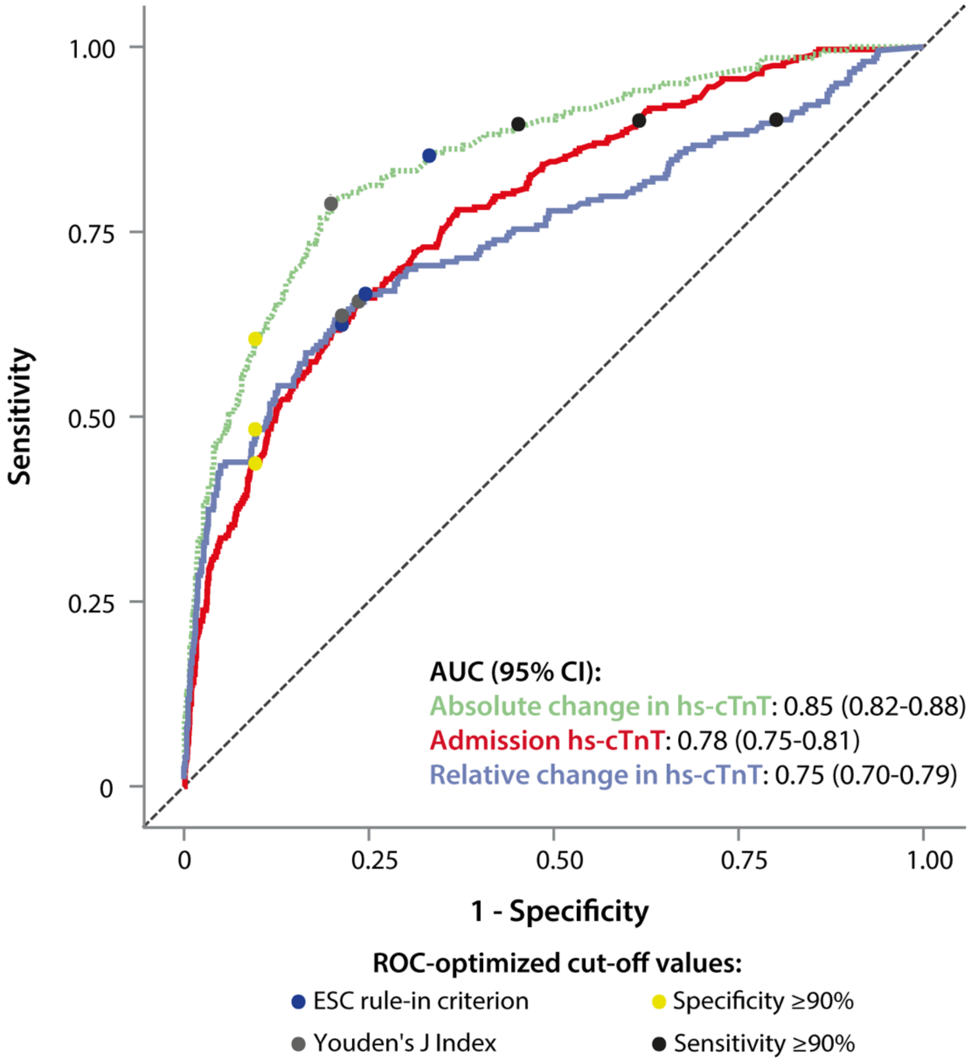

© 2024 Cyon L et al. *JAMA Network Open.*
