# Supplementary material for: Acute Kidney Injury and High-Sensitivity Cardiac Troponin T Levels in the Emergency Department
Source: JAMA Netw Open. 2024 Aug 30;7(8):e2419602. doi: 10.1001/jamanetworkopen.2024.19602 (PMC11365000; doi:10.1001/jamanetworkopen.2024.19602)
Supplement: Supplement 2. — Data Sharing Statement [file jamanetwopen-e2419602-s002.pdf]

Data Sharing Statement

Cyon. Acute Kidney Injury and High-Sensitivity Cardiac Troponin T Levels in the Emergency Department. *JAMA Netw Open*. Published August 30, 2024.
doi:10.1001/jamanetworkopen.2024.19602

Data
Data available: No
